# The Relationship Between Atrial Fibrillation and Intestinal Flora With Its Metabolites

**DOI:** 10.3389/fcvm.2022.948755

**Published:** 2022-07-01

**Authors:** Dasheng Lu, Xinyue Zou, Hongxiang Zhang

**Affiliations:** ^1^Department of Cardiology, The Second Affiliated Hospital of Wannan Medical College, Wuhu, China; ^2^Vascular Diseases Research Center of Wannan Medical College, Wuhu, China

**Keywords:** gut microbiota, atrial fibrillation, short-chain fatty acids, lipopolysaccharide, bile acids, trimethylamine N-oxide

## Abstract

Atrial fibrillation (AF) is characterized by high morbidity and disability rate. The incidence of AF has rapidly increased due to increased aging population, causing a serious burden on society and patients. Therefore, it is necessary to determine the prevention and treatment of AF. Several studies have assessed the occurrence, development mechanism, and intervention measures of AF. The human gut has several non-pathogenic microorganisms forming the gut flora. The human gut microbiota plays a crucial role in the construction and operation of the metabolic system and immune system. Emerging clinical studies and basic experiments have confirmed that intestinal flora and its metabolites have a role in some metabolic disorders and chronic inflammatory diseases. Moreover, the gut microbiota has a role in cardiovascular diseases, such as hypertension and heart failure. However, the relationship between AF and gut microbiota is unclear. This review summarizes the relevant literature on the relationship between AF and intestinal flora with its metabolites, including Trimethylamine N-Oxide, short-chain fatty acids, lipopolysaccharide and bile acids. Therefore, this review may enhance further development of related research.

## Introduction

Atrial fibrillation (AF) is one of the most common cardiac arrhythmias in clinics. The baseline prevalence of AF is about 4.9% in the general population aged 60 years and above, based on the latest epidemiological survey. Moreover, a 6-year observation found that the cumulative new incidence rate of AF is 4.1% (about seven new AF cases per 1,000 people yearly) (1). The incidence of AF rapidly increases with age. AF significantly increases the risk of ischemic stroke (cerebral infarction) and cardiac insufficiency. AF reduces the end-diastolic volume due to the clinical and pathophysiological characteristics of AF, such as fast ventricular rate, atrioventricular dyssynchrony, and loss of atrial systolic pump function, leading to a decline in cardiac systolic function and heart failure. AF is usually associated with cardiac insufficiency, thus greatly reducing the quality of life and prognosis of patients (2). Moreover, AF increases the mortality, recurrence rate, and disability rate of ischemic stroke patients (3). The high incidence and disability rate of AF causes a serious burden on society and patients. Therefore, the prevention and treatment of AF are necessary. Several studies have assessed the occurrence, development mechanism, and intervention measures of AF.

The human gut is the habitat of several non-pathogenic microorganisms forming gut flora. The human gut microbiota plays a key role in the construction and operation of the metabolic system and immune system. For example, gut microbes can metabolize food cholines, forming trimethylamine (TMA), which can be further converted trimethylamine N-oxide (TMAO) by liver monooxygenase. Emerging clinical studies and basic experiments have confirmed that intestinal flora and its metabolites have a role in some metabolic disorders and chronic inflammatory diseases (4, 5). The relationship between gut microbiota/its metabolites and cardiovascular diseases has recently attracted much attention (6). The disturbance of intestinal flora promotes the occurrence and development of hypertension (7–9). A study used fecal content transplantation from hypertensive human donors to germ-free mice to demonstrate the direct blood pressure-raising effect of gut microbiota (7). TMAO, a key metabolite in the intestinal flora, can significantly prolong the blood pressure-raising effect of angiotensin II in rats (10). Moreover, choline food or its intestinal metabolite TMAO can aggravate the deterioration of cardiac function and myocardial fibrosis in mice with heart failure (11, 12). Besides TMAO, other metabolites of gut microbiota, such as short-chain fatty acids (SCFAs), lipopolysaccharide (LPS), and bile acids, can also communicate with the host through metabolism and immune system (13–17). Moreover, the relationship between AF and gut microbiota is unclear. This review summarizes the relevant literature on the relationship between AF and intestinal flora with its metabolites. Therefore, this review may enhance further development of related research in the future.

## The Relationship Between Intestinal Flora With the Incidence, Course and Type of AF

Recent studies revealed distinct microbiome features for AF (18–21). With regards to AF onset, Zuo et al. (2019) explored the relationship between intestinal flora dysbiosis and AF (18) using 50 AF cases and 50 matched controls for the detection of fecal intestinal flora. Results showed that the composition of intestinal flora in AF patients had significant changes compared with normal subjects, manifested as increased *Ruminococcus, Streptococcus*, and *Enterococcus*, and decreased *Faecalibacterium, Alistipes, Oscillibacter*, and *Bilophila* (18). As alterations in gut microbiota, microbial metabolite profiles were changed in both fecal and serum samples of AF patients (18). Moreover, Gut microbiota was associated with not only the onset of AF but also the duration of persistent AF. The same team evaluated the differences in gut microbiota among short-term AF (<12 months), long-term AF (>12 months), and the control group (non-AF) using metagenomic sequencing and metabolomics analysis (19). Results showed short-term AF and long-term AF patients shared many common disordered gut microbiota and metabolic features, which may occur in the early stage (19). However, unique alterations occurred with increasing AF duration. For instance, longer AF duration was associated with decreased abundance of *Butyricicoccus* and *Paraprevotella, and* increased abundance of *Blautia, Dorea*, and *Coprococcus* (19). *Coprococcus* has been linked to an increased risk for coronary heart disease (22), while *Butyricicoccus* is SCFAs-producing bacteria (23). Therefore, the pathology of persistent AF may be related to the imbalance of gut microbiota. This study suggests that early prevention of intestinal flora dysbiosis and metabolite alterations are necessary for early intervention of AF. In addition, gut microbiota may discriminate the type of AF (i.e., paroxysmal AF and persistent AF). Based on the above-mentioned technique, Zou Kun and colleagues also compared the gut microbiota differences among paroxysmal AF, persistent AF, and the general population (20). Results showed that paroxysmal AF and persistent AF had similar gut microbiome diversity, taxonomic profiles, microbial functions, and metabolic features (20). However, they showed that specific gut microbes are associated with serum choline. They also showed that serum choline is associated with left atrial diameter (20). These findings suggest that specific microbes may be involved in the metabolism of specific metabolites and promote AF progression. Notably, these findings were all based on Chinese cohorts. Recently, Tabata et al. confirmed the association between AF onset and gut microbiota in a Japanese cohort (21). They reported that gut microbiota richness and composition are changed in AF patients. For example, they showed that genera including *Parabacteroides, Lachnoclostridium, Streptococcus*, and *Alistipes* are increased in AF patients, while *Enterobacter* is decreased, due to the higher intake of n-3 polyunsaturated fatty acids and eicosadienoic acid (21), suggesting the effects of diet. The typical microbiome and metabolites for AF in different studies were summarized in Table 1.

**Table 1:** Representative gut microbiome and metabolites for AF in different studies.

**Author (year)**	**Subjects**	**Representative bacteria changed** **at the genus level**	**Typical metabolites**	**Conclusion**	**Ref**.
Zuo Kun (2019)	AF and controls	**Increase:** *Streptococcus, Enterococcus, Blautia, Dorea, Veillonella, Coprobacillus and Ruminococcus* **Decrease:** *Oscillibacter*	Increase: cytokines^*^ and cytolysin^*^; Decrease: SCFAs^*^.	AF is associated with disordered gut microbiota and microbial metabolite profiles.	(18)
Zuo Kun (2019)	Persistent AF < 12 momths, > 12 months and controls	**Increase (longer AF duration):** *Blautia, Dorea*, and *Coprococcus;* **Decrease (longer AF duration):** *Butyricicoccus* and *Paraprevotella*	Increase (longer AF duration): l-tryptophan and pyroglutamic acid; Decrease (longer AF duration): oleamide, niacin, indole, choline, 3-indoleacetic acid, and phosphohydroxypyruvic acid and SCFAs^*^.	Duration of persistent atrial fibrillation is linked to changes in gut microbiota and metabolic phenotypes	(19)
Zuo Kun (2020)	paroxysmal AF, persistent AF and controls	**Increase (persistent AF):** *Methylovulum and Holospora*	Increase: choline	Different types of AF exhibit a limited degree of microbialshift	(20)
Tabata Tokiko(2020)	AF patients and controls	**Increase:** *Parabacteroides, Lachnoclostridium, Streptococcus, Alistipes Butyricimonas and Dorea;* **Decrease:** *Enterobacter*	Increase: TMAO^*^	AF patients had altered gut microbiota composition	(21)
Huang Kang (2022)	AF patients underwent catheter ablation and controls	**Increase (1 months after ablation):** *Agathobacter, Subdoligranulum, Lachnospira, Prevotella, Lactobacillus, Streptococcus, Haemophilus, and Coprococcus;* **Decrease (1 months after ablation):** *Ralstonia, Weissella, and Dialister*	Increase (1 months after ablation): SCFAs^*^ and citrulline; Decrease (1 months after ablation): cytokines^*^ and oleanolic acid	Gut Microbiota and Metabolites in AF Patients Changed after Catheter Ablation	(24)

*AF, atrial fibrillation; SCFAs, short-chain fatty acids; TMAO, trimethylamine N-oxide.*

* *Indicates that the metabolite alteration is speculated in the study, based on other publications*.

Beside AF, treatment of AF is also associated with altered gut microbiome. A recent study compared gut microbiota composition in AF patients prior to catheter ablation (CA), 2 days post CA and 1 month post CA (24). Compared with preoperative condition, 6 bacterial genera (*Prevotella, Lactobacillus, Sutterella, Alistipes, Parabacteroides*, and *Streptococcus*) enriched and 3 genera (*Blautia, Ruminococcus_gnavus_group, and Anaerostipes*) remarkably decreased 2 days after CA. At 1 months following CA, the abundances of eight genera (*Agathobacter, Subdoligranulum, Lachnospira, Prevotella, Lactobacillus, Streptococcus, Haemophilus*, and *Coprococcus)* increased, and 3 genera (*Ralstonia, Weissella*, and *Dialister)* were depleted. Among the increased genera after CA, several are (e.g., *Agathobacter and Prevotella)* associated with production of *SCFAs* (25, 26), which have beneficial effects on cardiovascular diseases (27). While *Ruminococcus_gnavus_group, which decreased after CA*, is associated with inflammatory diseases (28). It seemed that CA enriches intestinal symbiotic bacteria and reduces pathogenic bacteria. Correspondingly, metabolites, such as oleanolic acid are downregulated after ablation, while citrulline is increased (24). This also emphasizes the crucial role of gut microbiota in AF pathogenesis.

Although most studies have shown that gut microbiota changes affect AF development, some researchers have shown an interplay between host genetics and the gut microbiome. For instance, Xu et al. conducted bidirectional Mendelian randomization analyses using a Chinese population (1,475 subjects) and found that AF can induce the abundance of some specific gut microbiota. They also demonstrated that AF enriches the abundance of *Burkholderiales* and *Alcaligenaceae* and reduces the abundance of *Lachnobacterium, Bacteroides coprophilus*, and *Barnesiellaceae* (29). However, the specific causal relationship between AF and intestinal flora should be further assessed to enhance the prevention and treatment of AF and cardiovascular diseases in the future.

## Relationship Between Gut Microbiota Metabolites and AF

### Lipopolysaccharide

Lipopolysaccharide (LPS) is a cell wall component of gram-negative bacteria. Tight junctions between colonic epithelium are destroyed under pathological conditions, and thus LPS enters the systemic circulation, leading to excessive inflammatory response damages (30, 31). Both a healthy diet and balanced gut microbiota are crucial to maintain an intact gut. For instance, high-fat diet results in gut microbial dysbiosis and LPS elevation (32). The gut LPS can damage gut barrier integrity (33), contributing to a “leaky” gut and chronic inflammation. The intestinal barrier is destructed in germ-free mouse, while colonization with *Lactobacillus* improves the intestinal barrier (34). It is reported that broad-spectrum antibiotics reduces expression of tight junction proteins in mouse intestine, while supplementation of *Lactobacillus* GG and tributyrin mitigated antibiotic-induced intestinal damage (35).

Daniele Pastori et al. analyzed the relationship between the intestinal flora metabolite LPS and major adverse cardiovascular events (MACE) in AF patients and the effect of adhering to the Mediterranean diet on LPS and MACE. This prospective, single-center study included 912 AF patients who had been anticoagulated with a vitamin K antagonist (average age; 73.5 years). The study found that the LPS level was higher in the group with MACE than in the group without MACE. Moreover, they showed that log-LPS value is a predictor of MACE occurrence. Further analysis showed that LPS is positively associated with platelet activation levels. However, results showed that Mediterranean diet rich in fruits and legumes can reduce blood LPS levels (36).

Zhang et al. recently conducted fecal microbiota transplantation from aged rats to young hosts to assess gut microbiota and age-related AF (37). They showed that the young hosts had increased LPS and glucose levels in circulation and up-regulated NOD-like receptor protein (NLRP)-3 inflammasome. Also, the young host had enhanced atrial fibrosis and AF susceptibility (37). Interestingly, the aged rats colonized with youthful microbiota had lower NLRP3-inflammasome activity and AF susceptibility (37). Cross-sectional clinical studies also showed that LPS is positively correlated with age (37). This study demonstrated that the gut mirobiota-atrial NLRP3 inflammasome axis could be a rational therapeutic target for AF. Of note, gut dysbiosis also contributes to intestinal barrier damage and impaired glucose tolerance (37), which may cause an increase in AF susceptibility as well. A latest metagenomic data-analysis showed that microbes involved in LPS synthesis are enriched in the intestinal tract of AF patients. Furthermore, LPS synthesis function of AF patients is up-regulated by encoding the LPS synthase gene (38). Alterations in LPS-producing bacteria are involved in the AF pathogenesis (38).

## Trimethylamine N-Oxide (TMAO)

TMAO is a key product of gut microbiota. It is associated with various diseases, including Alzheimer's disease (39), chronic kidney disease (40), ischemic stroke (41), heart failure (42), and acute coronary syndrome (43). Yu et al. showed that the intestinal flora metabolite TMAO can increase atrial electrical instability in normal canines using animal experiments (44). They also showed that TMAO improves AF inducibility and can also exacerbates the increased neural activity and the induction of AF via atrial pacing, possibly because TMAO stimulates the release of inflammatory factors and activates the p65 NF-κB signaling pathway (44). Gong et al. assessed the relationship between intestinal flora product TMAO and thrombosis in AF patients (45). The study enrolled patients with valvular AF and divided them into two groups: thrombotic group and non-thrombotic group. They showed that the baseline characteristics between the two groups were not significantly different. The researchers also measured serum TMAO, choline levels, and platelet function in both groups. The final analysis showed that the TMAO level was significantly higher in the thrombus group than in the non-thrombotic group. Moreover, the platelet function was over-activated in the thrombus group (45). Furthermore, a bivariate regression analysis confirmed that serum TMAO levels could predict thrombosis risk. Therefore, serum TMAO level detection can be used for accurate prevention of embolic events in AF patients (45). Current research suggests that TMAO can be used as a predictive tool for diseases, such as AF, and is thus a key target for the development of new treatments (46). Several studies, however, did not find a correlation of TMAO with atherosclerosis (47) or systemic activation of coagulation (48). The relationship between AF and TMAO needs more investigations.

### Short-Chain Fatty Acids (SCFAs)

SCFAs are the main metabolites produced by the colonic flora decomposing dietary fiber. SCFAs mainly comprise acetate, propionate, and butyrate. SCFAs exert immunoregulatory effect on the host by acting on intracellular specific G-protein-coupled receptors (GPR)41 and histone deacetylase (49, 50). SCFAs mitigate system inflammation and regulate high blood pressure (49, 50). SCFAs can counteract the inflammatory response induced by LPS (51). A recent study demonstrated that the butyrate-producer *Roseburia intestinalis* improves intestinal barrier function, counteracts endotoxemia and protect from atherosclerosis (52), indicating that SCFAs may prevent cardiovascular disease progression through barrier protection. SCFAs theoretically have a protective effect on AF development. A few studies have assessed the relationship between SCFAs and AF. For instance, Zhang et al. reported that AF patients have significantly decreased microbial genes involved in SCFA-related synthesis, based on a metagenomic data-mining analysis in a northern Chinese population (53). Nevertheless, further studies are needed to assess the role of gut bacteria producing SCFAs in AF.

### Bile Acids

Cholesterol plays a pivotal role in cardiovascular disorders, including atherosclerosis. Using germ-free hyperlipidemia mouse models, Kiouptsi et al. confirmed the cholesterol-lowering effect of the gut microbiota at normal diet conditions (54). Primary bile acids are synthesized directly from cholesterol exclusively in the liver, and then are secreted into the bile and intestine. Gut microbiome can metabolize primary bile acids into secondary bile acids. Bile acids are extensively modified by the microbiome (55, 56). Bile acids act as metabolic regulator of lipid and glucose metabolism (57, 58). Bile acids also exert anti-inflammatory effects by inhibiting NF-kB signaling pathways (59) and NLRP3- inflammasome activities (60). Besides, AF patients have altered serum bile acid composition. Furthermore, serum concentration of chenodeoxycholic acid (CDCA), a primary bile acid, is significantly higher in persistent AF than in paroxysmal AF. Serum CDCA level is positively associated with the left atrial low voltage area in AF patients (61). Besides, CDCA promotes apoptosis of mouse atrial myocytes. CDCA enhances the structural remodeling of AF (61). Alonso et al. showed that glycolithocholate sulfate and glycocholenate sulfate, two conjugated bile acids, are significantly associated with the risk of AF in African-American patients (62). The research team also showed that glycocholenate sulfate may be related to AF incidence (63). Rainer et al. showed that two bile acids, taurocholic acid (TCA) and ursodeoxycholic acid have different effects on human atrial myocardium. For instance, high TCA concentrations induce arrhythmias in adult human atria, while non-ursodeoxycholic acid is associated with clinical AF. Moreover, AF patients have significantly reduced serum levels of ursodeoxycholic acid conjugates, indicating the potentially protective effect of ursodeoxycholic acid (64). Lithocholic acid, a secondary bile acid, can prevent doxazosin-induced apoptosis in cardiomyocytes by suppressing EphA2 phosphorylation (65), and thus might help in the treatment of AF and heart failure.

## Conclusion

**Figure 1:**
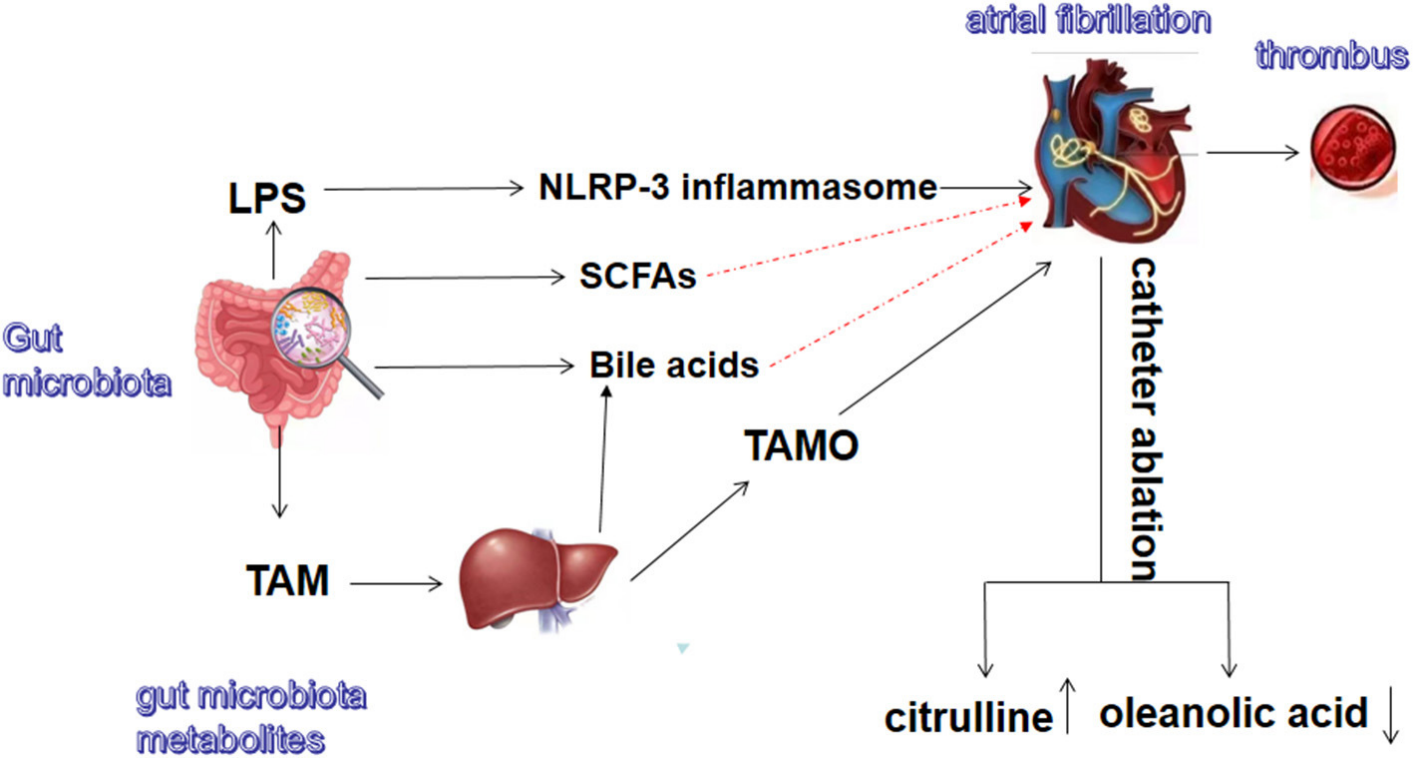
Gut microbiota and metabolites related to atrial fibrillation. TMA, trimethylamine; TMAO, trimethylamine N-oxide; LPS, lipopolysaccharide; SCFAs, short-chain fatty acids. The red dash lines indicate these metabolites might exert protective effect on atrial fibrillation.

In summary, gut microbiota and its metabolites are closely related to AF (Figure 1). Notably, the pathogenesis of AF is multifaceted and may involve neuromodulation, triggering mechanisms, atrial remodeling (atrial cardiomyopathy), and atrial electrical remodeling. Moreover, there are diverse treatment methods for AF. Recent advances in the treatment of AF include the clinical application of rhythm control, such as radiofrequency ablation. Catheter radiofrequency ablation can significantly improve the quality of life and prognosis of AF patients compared with traditional drug therapy (66, 67). The new oral anticoagulants (dabigatran, rivaroxaban, and apixaban) have many advantages (68). AF is a complex arrhythmia disease that is difficult to fully explain or overcome through a single pathway. Although many studies have shown that intestinal flora is related to AF, further research is needed to confirm this. Most studies have only found an association between gut microbiota disturbances and AF, but the causal relationship requires further verification. Additionally, no study has reported clinical treatment approaches targeting gut microbiota and its metabolites in AF. The current modulation methods for the intestinal flora mainly include fecal transplantation, antibiotics, and the use of probiotics. Dietary habits and physical activity may also alter gut microbiota. Sharafedtinov et al. reported that supplementation of probiotic cheese in obese hypertensive patients may help reduce body mass index and arterial blood pressure (69). A recent clinical randomized controlled trial also showed that probiotic and commensal bacteria supplementation in pre-diabetic patients can improve the prevalence of metabolic syndrome. For instance, probiotic supplementation can significantly reduce hypertension (70). However, further studies are needed to investigate the role of these treatments in AF.

## Author Contributions

DL: conception, writing, and editing XZ: writing and construction. HZ: writing and editing. All authors have a significant contribution to this work and contributed to the article and approved the submitted version.

## Funding

This work was supported by grant from the National Natural Science Youth Foundation of China (81800445) and Cultivation Fund for Key Scientific Research Project of Wannan Medical College (WK2018ZF10).

## Conflict of Interest

The authors declare that the research was conducted in the absence of any commercial or financial relationships that could be construed as a potential conflict of interest.

## Publisher's Note

All claims expressed in this article are solely those of the authors and do not necessarily represent those of their affiliated organizations, or those of the publisher, the editors and the reviewers. Any product that may be evaluated in this article, or claim that may be made by its manufacturer, is not guaranteed or endorsed by the publisher.
